# Species-Dependent Antifungal Profiles of Chitosan and Sulphated Polysaccharide-Rich Extract from *Jania pedunculata* var. *adhaerens*

**DOI:** 10.3390/md24090296

**Published:** 2026-08-24

**Authors:** Miguel Valverde-Urrea, Jose Defez-Pérez, Maria Francisca Colom-Valiente, Marc Terradas-Fernández, Luis V. López-Llorca, Federico Lopez-Moya

**Affiliations:** 1 Department of Marine Sciences and Applied Biology, University of Alicante, Aptdo. Correos 99, 03080 Alicante, Spainmarc.terradas@ua.es (M.T.-F.); lv.lopez@ua.es (L.V.L.-L.); 2 Department of Microbiology and Plant Production, Miguel Hernández University, 03550 Alicante, Spain; colom@umh.es

**Keywords:** *Jania pedunculata* var. *adhaerens*, sulphated polysaccharides, chitosan, antifungal activity, pathogenic yeasts, *Candida auris*, marine biopolymers

## Abstract

Yeast infections are becoming an increasing public health concern, mainly due to the spread of opportunistic species and the emergence of strains resistant to commonly used antifungal drugs. Marine resources are a promising source of bioactive compounds, including polysaccharides and other biopolymers with potential antifungal applications. In this study, a sulphated polysaccharide-rich extract was obtained from the red alga *Jania pedunculata* var. *adhaerens* and chemically characterized. Its antifungal activity was compared with that of a commercial chitosan formulation against clinically relevant yeasts, including species of *Candida*, *Cryptococcus*, *Clavispora*, *Naganishia* and *Trichosporon*. Growth kinetics were monitored in liquid medium over 24 h, and antifungal activity was evaluated through growth rate analysis, growth inhibition at 20 h and susceptibility clustering. The sulphated polysaccharide-rich extract showed moderate but consistent growth inhibition, with the strongest effects observed at 5 mg mL^−1^. Maximum growth inhibition reached 60.9% in *Cryptococcus deuterogattii* and 59.8% in *Candida albicans*, although no complete inhibition was observed within the tested concentration range. Chitosan showed a stronger antifungal effect, with minimal inhibitory concentration (MIC) values between 10 and 20 µg mL^−1^ in several species and maximum inhibition values above 80% in the most susceptible yeasts. However, *C. albicans* showed marked resistance to chitosan, with inhibition below 12%. K-means clustering confirmed distinct susceptibility profiles between treatments, supporting a species-dependent response. Overall, these results highlight marine-derived biopolymers as promising antifungal candidates and show that chitosan and algal sulphated polysaccharides produce distinct, species-dependent antifungal profiles.

## 1. Introduction

Fungal infections are an increasing global health problem, with more than 1.5 million deaths reported annually [1,2]. Although more than 1.5 million fungal species are estimated to exist, only ca. 600 can infect humans [3,4]. Among these, *Aspergillus*, *Candida* and *Cryptococcus* spp. are the most clinically relevant [5]. Furthermore, *Candida* spp. are responsible for the majority of human mycoses [6,7,8].

Infections caused by *Candida* species are of particular concern due to their high incidence and associated healthcare costs. These infections range from superficial or mucosal candidiasis to invasive disease, including candidemia, which refers to the presence of *Candida* in the bloodstream. The estimated incidence of candidiasis ranges from 2 to 14 cases per 100,000 inhabitants, while candidemia affects up to 250,000 people annually worldwide [9]. Among these species, *Candida auris* has emerged as a major pathogenic yeast threat due to its resistance to multiple antifungal drug classes, including polyenes, azoles and echinocandins [10,11]. *C. auris* has been reported in more than 30 countries and is associated with high mortality rates and environmental persistence, which facilitates horizontal transmission [8,12,13]. Beyond *Candida*, other opportunistic yeasts, particularly species of *Cryptococcus*, *Clavispora*, *Naganishia* and *Trichosporon*, are also increasingly recognized as clinically relevant pathogens.

Infections caused by *Cryptococcus* species (cryptococcosis) mainly affect immunocompromised patients, including those with HIV infection, diabetes or lymphocytopenia, as well as individuals undergoing treatment with corticosteroids or immunosuppressive drugs [14,15]. Other emerging or opportunistic yeasts also represent a growing concern, such as *Clavispora lusitaniae*. This yeast can cause severe infections such as meningitis and peritonitis and shows resistance to commonly used antifungal agents such as fluconazole and amphotericin B [16]. Although *Naganishia albida* is not typically pathogenic, increasing cases of infection in immunocompromised patients have been reported [17,18]. Similarly, *Trichosporon cutaneum* is considered an emerging opportunistic pathogen, often associated with biofilm formation on medical devices, which facilitates cross-infections [19].

Despite the availability of antifungal drugs, the emergence of resistant strains is increasing and represents a major clinical challenge [20]. This situation highlights the need to identify new antifungal compounds and alternative therapeutic strategies [21].

Marine resources constitute a promising source of bioactive molecules due to their high diversity, abundance and unique chemical structures [22,23,24,25]. Among these resources, marine algae have attracted considerable attention. Recent reviews have highlighted marine-derived compounds as promising antimicrobial compounds, particularly in the context of drug-resistant microorganisms [26]. Several studies have demonstrated the antifungal activity of algal extracts against human pathogenic fungi. For example, [27] reported growth inhibition of *Candida* species using polar extracts from *Saccharina japonica*, *Laminaria japonica*, *Undaria pinnatifida*, *Palmaria palmata* and *Eisenia bicyclis*. In addition, apolar extracts from *Jania pedunculata* var. *adhaerens* have shown antifungal activity against *C. albicans* [28].

Among algal-derived compounds, sulphated polysaccharides (PS) have gained particular interest due to their wide range of biological activities, including antifungal properties [29,30]. These compounds are characterized by the presence of sulphate groups (SO_3_^−^) attached to sugar residues. Algae represent one of the main natural sources of PS, including fucoidans, carrageenans, galactans and agar. Sulphated polysaccharides from marine macroalgae are increasing in relevance in pharmacology, although their antimicrobial potential remains strongly dependent on their algal origin, chemical composition and degree of sulphation [31]. The composition of these polysaccharides depends on the algal group, with sulphated galactans predominating in red algae [32]. Although some studies have reported antifungal effects of PS, such as the alteration of fungal cell morphology by carrageenans in *Aspergillus fumigatus* [33], their activity against clinically relevant yeasts such as *Candida* and *Cryptococcus* remains poorly characterized [33,34].

Articulated coralline algae are dominant along the Alicante coast and show high tolerance to environmental stress [35,36]. Among them, *Jania pedunculata* var. *adhaerens* is abundant and easily collected, forming conspicuous belts along the coastline [37]. This species has shown high polysaccharide extraction yields and relevant sulphate content [32], making it a suitable candidate for antifungal studies.

In addition to algal-derived compounds, other marine biopolymers such as chitosan have shown strong antifungal activity [38,39]. Chitosan, the N-deacetylated form of chitin, exhibits favourable physicochemical properties, including solubility in mildly acidic conditions and a positive charge that enhances its interaction with microbial cells [40]. It is mainly obtained from seafood biomass, contributing to circular economy strategies and reducing environmental impact [41,42].

It has been reported that chitosan exerts fungistatic effects on *C. albicans* at low concentrations (2.5–10 µg mL^−1^), likely related to membrane disruption targeting ARL1, ion imbalance and metabolic alterations [43,44]. Other mechanisms, such as metal chelation, enzyme inhibition and interaction with DNA, have also been proposed [44]. In addition, chitosan has shown fungicidal activity against other clinically relevant yeasts, including *C. glabrata*, *C. parapsilosis* and *Cryptococcus* species [45], even in fluconazole-resistant strains [46].

In this context, the present study evaluates the antifungal activity of a sulphated polysaccharide-rich extract from a new algal source, *J. pedunculata* var. *adhaerens*, against selected species of *Candida* and *Cryptococcus*, while also characterizing the antifungal activity of crustacean-derived chitosan across a broader panel of clinically relevant yeast species and genera. This approach allowed us to characterize species-dependent susceptibility to both marine-derived biopolymers under the same experimental conditions and to identify antifungal profiles with potential relevance for antifungal applications.

## 2. Results

### 2.1. Algae Identification and Characterization

The collected algal biomass was morphologically identified as *Jania pedunculata* var. *adhaerens* based on its characteristic dichotomous thallus, pale pink-lilac coloration and articulated calcified structure (Figure S1). The specimens presented an approximate length of 5 cm, with calcified intergenicula measuring 80–120 µm in diameter and lengths approximately 5–6 times greater than their width. In addition, dichotomy angles were consistently greater than 30°, in agreement with the diagnostic morphological features previously described for *J. pedunculata* var. *adhaerens* (Figure S1). Molecular identification based on COI sequencing further supported this classification, showing ≥99% similarity with reference sequences available in the NCBI database.

### 2.2. Jania pedunculata *var.* adhaerens Yielded a Sulphated Polysaccharide-Rich Extract with Characteristic FTIR Signatures

The sulphated polysaccharide-rich extract yielded 3.7 ± 0.3% (*w*/*w*) relative to the initial dry biomass. The extract was predominantly composed of carbohydrate fractions, with insoluble polysaccharides accounting for 56.23% and soluble polysaccharides for 20.24% of the total extract. Lipids (10.67%) and proteins (2.25%) were present in lower proportions.

Elemental analysis indicated the presence of mineral components, including Ca (2.5%), Mg (0.41%), K (0.028%), Na (0.078%) and S (1.87%).

Monosaccharide analysis revealed a heterogeneous composition dominated by arabinose (62.1%), followed by xylose (17.1%) and fructose (16.0%), while rhamnose was detected at lower proportions (4.7%). This heterogeneous monosaccharide profile is consistent with the structural complexity commonly described for sulphated polysaccharides from red macroalgae. Galactose was not quantified because an analytical standard was not available.

**Figure 1:**
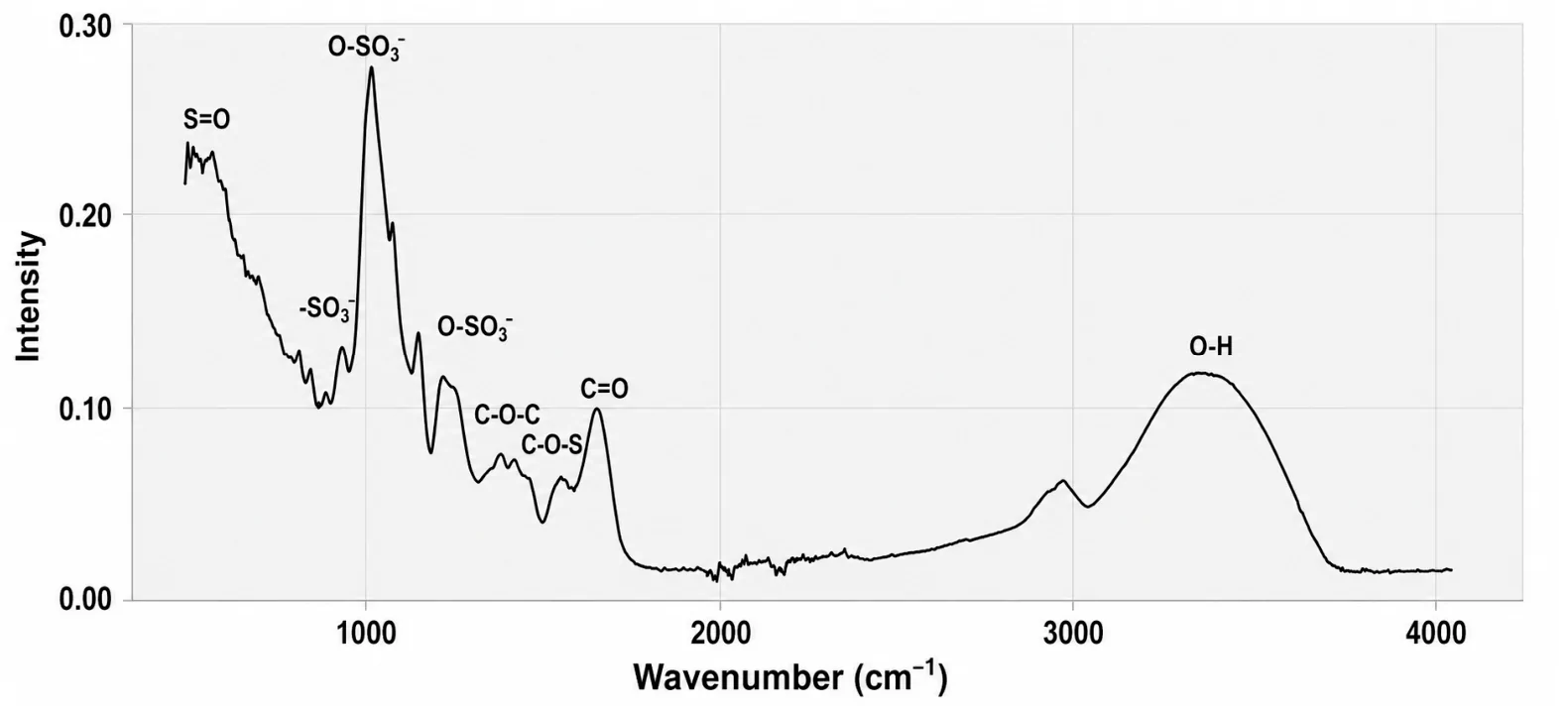
FTIR analysis of the crude sulphated polysaccharide-rich extract obtained from *J. pedunculata* var. *adhaerens*.

FTIR analysis revealed characteristic absorption bands associated with sulphated polysaccharides (Figure 1). Bands detected between 500 and 600 cm^−1^ and at 931 cm^−1^ were associated with sulphate groups (S=O and SO_3_^−^), while signals between 1220 and 1270 cm^−1^ corresponded to sulphate ester bonds (O–SO_3_^−^), which are typical features of sulphated polysaccharides from red algae [47,48]. In addition, the band at 1644 cm^−1^ was related to C=O vibrations, whereas signals observed between 2833 and 2963 cm^−1^ and 3412–3433 cm^−1^ were attributed to C–H and O–H stretching vibrations, respectively, confirming the polysaccharidic nature of the extract [48,49]. Sulphate groups were detected using a turbidimetric method, estimating a sulphate content of 11.3% of the dry extract.

### 2.3. Chitosan Showed Strong but Species-Dependent Antifungal Activity Against Pathogenic Yeasts

Chitosan showed a marked antifungal effect that was both concentration- and species-dependent against most of the yeast species tested (Figures S2–S4). Growth kinetics and growth rate parameter (r) analyses revealed a progressive reduction in fungal growth as chitosan concentration increased, with significant differences detected in all species (ANOVA, *p* < 0.05; Figures S2–S10, Tables S1–S3). However, susceptibility patterns varied considerably among species, ranging from highly sensitive to moderately resistant responses.

**Figure 2:**
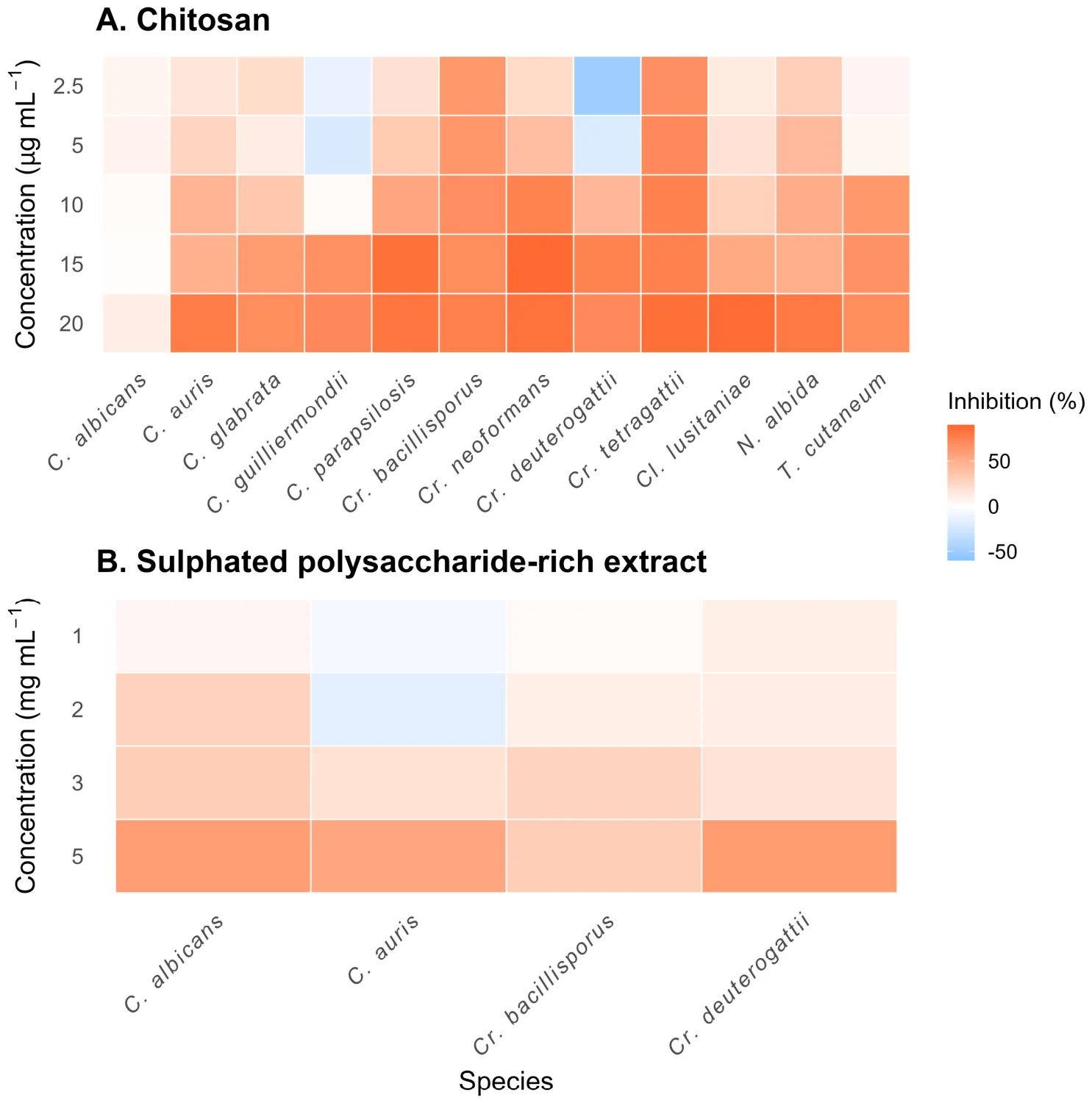
Heatmap representation of the antifungal activity of (**A**) an aqueous solution of chitosan and (**B**) the sulphated polysaccharide-rich extract from *J. pedunculata* var. *adhaerens* against different yeast species at increasing concentrations. Colours represent the percentage of growth inhibition relative to the untreated control, ranging from stimulation (blue tones, negative values) to strong inhibition (orange-red tones, positive values).

The highest antifungal activity of chitosan was observed in *Cr. deneoformans*, *Cl. lusitaniae*, *Cr. tetragattii*, *C. parapsilosis* and *N. albida*, which reached maximum inhibition values above 80% and MIC values between 10 and 20 µg mL^−1^ (Table 1, Figure 2). However, *C. albicans* showed very low sensitivity to chitosan, with inhibition values remaining below 12% even at the highest concentration tested, despite a significant reduction in the growth rate parameter. Similarly, *T. cutaneum* and *C. guilliermondii* maintained detectable growth at all tested concentrations, indicating lower susceptibility to the treatment.

Interestingly, some species, particularly *C. guilliermondii* and *Cr. deuterogattii*, showed growth stimulation at low chitosan concentrations before inhibition occurred at higher doses. Overall, growth inhibition generally increased in a concentration-dependent manner, reaching values between 69% and 88% in the most susceptible species.

### 2.4. Sulphated Polysaccharide-Rich Extract Showed Moderate and Species-Dependent Growth Inhibition

The sulphated polysaccharide-rich extract was evaluated against four clinically relevant yeast species and showed moderate, species-dependent antifungal activity (Figures S11–S13). Growth rates differed significantly among treatments (one-way ANOVA, *p* < 0.05, Tables S4 and S5), although the magnitude of the effect was lower compared to chitosan.

No complete inhibition was observed within the tested concentration range (1–5 mg mL^−1^), whereas chitosan showed antifungal activity at substantially lower concentrations (2.5–20 µg mL^−1^). MIC values for the sulphated polysaccharide-rich extract were therefore consistently above 5 mg mL^−1^ (Table 1, Figure 2).

Maximum inhibition values reached 60.9% in *Cr. deuterogattii* and 59.8% in *C. albicans* at 5 mg mL^−1^, whereas other species, such as *Cr. bacillisporus*, showed lower inhibition levels, not exceeding 30%.

As observed for chitosan, low concentrations occasionally resulted in slight growth stimulation. This effect was particularly evident in *C. auris*, where negative inhibition values were recorded at 1 and 2 mg mL^−1^.

### 2.5. K-Means Clustering Revealed Distinct Susceptibility Profiles Between Treatments

**Figure 3:**
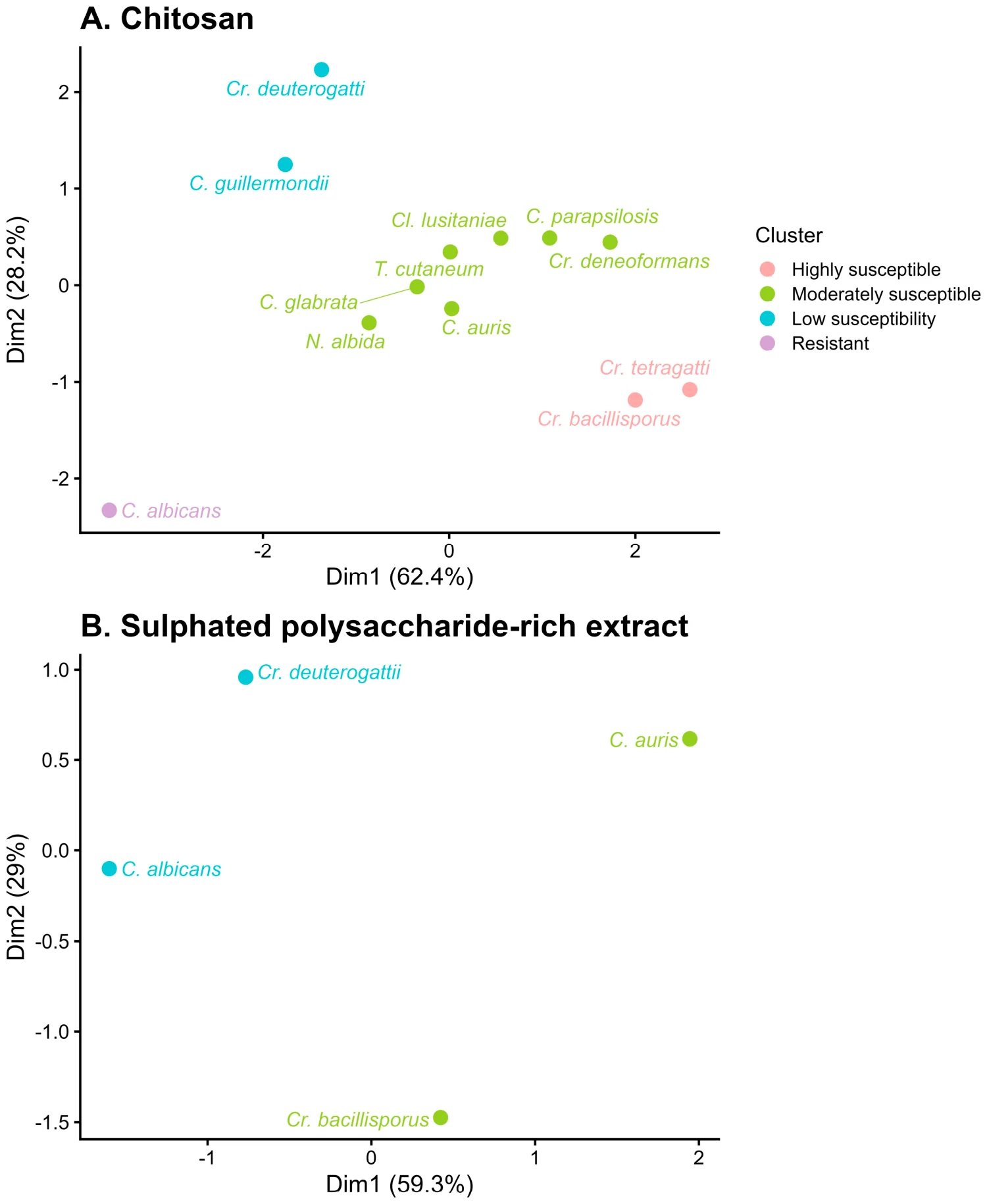
K-means clustering analysis of the antifungal response patterns of different yeast species treated with (**A**) an aqueous solution of chitosan and (**B**) the sulphated polysaccharide-rich extract obtained from *Jania pedunculata* var. *adhaerens*. Species were grouped according to their inhibition profiles across the tested concentration ranges.

To further explore species-specific responses to both treatments, a K-means clustering analysis was performed based on inhibition profiles across concentrations (Figure 3).

For chitosan-treated yeast, the analysis revealed a clear separation of species according to their sensitivity to chitosan. Highly sensitive species, mainly within the genus *Cryptococcus*, showed strong inhibition even at low concentrations, whereas other species displayed a more gradual, dose-dependent response. In contrast, *Candida albicans* showed minimal inhibition across all concentrations and was clearly separated from the rest of the dataset. Intermediate patterns were observed in species such as *C. auris* and *C. parapsilosis*, which exhibited a progressive increase in inhibition with increasing chitosan concentration.

Some species did not follow a simple dose–response pattern. In particular, *Cr. deuterogattii* and *C. guilliermondii* showed a biphasic behaviour, with stimulation of growth at low concentrations followed by inhibition at higher doses. This pattern contributed strongly to their position in the multivariate space and highlights the variability in the response to chitosan among yeast species.

For the sulphated polysaccharide-rich extract, clustering showed a more limited differentiation between species, with most responses grouped within a narrower range of inhibition values. Although inhibition increased with concentration, the overall effect was lower compared to chitosan, and no species reached complete inhibition within the tested range.

The first principal component explained more than 60% of the total variance, indicating that the main trends in the data were well captured by the analysis. Overall, clustering supported the existence of distinct response patterns between species and confirmed the stronger and more variable antifungal effect of chitosan compared to the sulphated polysaccharide-rich extract.

## 3. Discussion

The extraction yield of the sulphated polysaccharide-rich extract obtained from *J. pedunculata* var. *adhaerens* (3.7%) was consistent with the value previously reported by Hentati et al. [32] (4.55%). This close agreement was observed despite the additional washing steps applied in the present study to remove pigments, phenolic compounds, proteins, and salts, which were intended to improve the purity and suitability of the extract for subsequent chemical and biological characterization.

Despite the lower yield, the sulphated polysaccharide-rich extract showed a consistent inhibitory effect against the four yeast species evaluated (*C. albicans*, *C. auris*, *Cr. deuterogattii* and *Cr. bacillisporus*) at 5 mg mL^−1^. This is the first study reporting antifungal activity of a sulphated polysaccharide-rich extract from *J. pedunculata* var. *adhaerens* against clinically relevant yeasts. However, the observed effect was moderate compared to chitosan, suggesting a lower intrinsic antifungal potency or a different mode of action. The presence of sulphate groups and sulphur content indicates that these compounds are likely sulphated polysaccharides, potentially belonging to the group of sulphated galactans typically found in red algae, although further structural characterization would be required to confirm their precise nature.

The mechanism underlying the antifungal activity of these polysaccharides remains unclear. Previous studies have reported alterations in fungal cell wall composition following exposure to algal polysaccharides, including reductions in β-1,3-glucan and chitin content [33]. In addition, sulphated polysaccharides have been associated with DNA damage, receptor interactions and metabolic disruption in microbial systems [50,51]. Their ability to interfere with biofilm formation and gene expression has also been described [52]. Although these mechanisms have mainly been reported in bacteria or filamentous fungi, they could also contribute to the inhibitory effects observed in yeasts. Accordingly, the antifungal activity reported here should be interpreted specifically for the sulphated polysaccharide-rich extract obtained from *J. pedunculata* var. *adhaerens* and should not be generalized to sulphated polysaccharides as a whole.

Recent studies also support the finding that marine-derived sulphated polysaccharides can show antifungal activity, although this activity is highly dependent on the algal source, composition and target organism. For example, carrageenan-rich extracts from red algae have been reported to affect fungal growth and morphology, while fucoidan extracts from brown algae showed species-dependent activity against yeasts, including *Candida* spp. [53,54,55,56,57]. These studies agree with our results, where the sulphated polysaccharide-rich extract from *J. pedunculata* var. *adhaerens* produced species-dependent inhibition of pathogenic yeast growth, supporting the need for further studies on red algal polysaccharides against pathogenic yeasts.

In contrast, chitosan exhibited strong antifungal activity across most species, with low MIC values (10–20 µg mL^−1^), confirming its broad antifungal activity across the tested species. This result is consistent with previous studies describing chitosan as a potent antifungal agent in both yeast and filamentous fungi [43,45]. The antifungal activity of chitosan has been attributed to its cationic nature, which enables interaction with negatively charged membrane phospholipids, leading to membrane disruption and increased permeability [58,59,60]. In addition, transcriptional changes affecting cell wall synthesis and stress response pathways have been reported [61,62,63].

Differences in antifungal activity between studies may be explained by experimental conditions. Nutrient availability has been shown to modulate sensitivity to chitosan, with carbon and nitrogen limitation increasing antifungal susceptibility [45]. The relatively high nutrient content of the medium used in this study may therefore explain the reduced sensitivity observed in *Candida albicans*, which was the most resistant species.

The physicochemical properties of chitosan, including molecular weight and degree of deacetylation, also play a key role in its antifungal activity. In this study, chitosan with a relatively high molecular weight (70 kDa) and a high degree of deacetylation (90.1%) was used, both of which have been associated with increased biological activity [64,65]. Moreover, fungal sensitivity to chitosan depends on membrane composition and cell wall structure, particularly the glucan-to-chitin ratio and fatty acid profile [58,63]. The higher resistance of *C. albicans* observed in this study may therefore be related to its membrane composition, which is enriched in saturated fatty acids [66].

Interestingly, *Candida auris* growth was almost completely inhibited by chitosan at 20 µg mL^−1^, highlighting its potential relevance as an alternative antifungal agent against this multidrug-resistant pathogen [67,68]. Given the limited therapeutic options currently available for *C. auris*, these results support further exploration of chitosan-based strategies. Although direct comparisons with conventional antifungals were not performed in this study, the observed response supports further evaluation of chitosan-based strategies against this multidrug-resistant pathogen. Moreover, chitosan can be obtained from marine by-products such as crustacean waste, making it an environmentally friendly biopolymer aligned with circular economy approaches.

Another relevant observation was the stimulation of growth at low concentrations of both chitosan and polysaccharides in some species. This effect may be related to hormetic responses or to the antioxidant properties of chitosan [69], which could enhance cell survival under mild stress conditions.

A key finding of this study is the differential response of yeast species to chitosan and sulphated polysaccharides; for instance, *C. albicans* showed low susceptibility to chitosan under the tested conditions. The sulphated polysaccharide-rich extract produced a moderate inhibitory effect at the concentrations evaluated. These responses should be interpreted within the different concentration ranges evaluated for each treatment rather than as directly equivalent levels of antifungal activity. This contrast likely reflects differences in their mechanisms of action. Chitosan, as a cationic polymer, primarily targets membrane integrity, whereas sulphated polysaccharides, being anionic, may interact differently with the cell surface and affect biofilm formation or cellular metabolism [52,61]. These complementary modes of action suggest that combined treatments could enhance antifungal efficacy, an approach that warrants further investigation.

Future studies should explore the influence of nutrient availability on antifungal activity, as well as evaluate these compounds under conditions more closely resembling physiological environments. In addition, testing drug-resistant strains and assessing potential synergistic effects between T8 chitosan and algal sulphated polysaccharides could provide valuable insights into their applicability as alternative or complementary antifungal strategies.

## 4. Materials and Methods

### 4.1. Yeast Strains

The antifungal activity of the sulphated polysaccharide-rich extract was tested against the following species: *Candida albicans*, *C. auris*, *Cryptococcus bacillisporus* and *Cr. deuterogattii*. For chitosan assays, a broader set of species was used: *Candida albicans*, *C. auris*, *C. glabrata*, *C. guilliermondii*, *C. parapsilosis*, *Cryptococcus bacillisporus*, *Cr. deuterogattii*, *Cr. deneoformans*, *Cr. tetragattii*, *Clavispora lusitaniae*, *Naganishia albida* and *Trichosporon cutaneum*. All yeast strains were kindly provided by Dr. Colom-Valiente from the Plant Production and Microbiology Laboratory (Miguel Hernández University, Spain).

### 4.2. Chitosan Formulation

An aqueous solution of chitosan was prepared from commercial chitosan powder (T8s, Biolog Hepe, Landsberg, Germany). The chitosan used had a medium molecular weight (70 kDa) and a degree of deacetylation of 90.1%. The powder was dissolved in 0.25 M hydrochloric acid (HCl), and the pH was adjusted to 5.60 [58]. The solution was dialyzed for 48 h using a cellulose membrane (12–15 kDa cut-off; Sigma-Aldrich, St. Louis, MO, USA) to remove salts. Finally, the solution was sterilized at 121 °C for 20 min. The aqueous solution of chitosan was stored at 4 °C until use.

### 4.3. Algae Biomass Collection and Morphological and Molecular Identification

Algal biomass was collected at Agua Amarga beach (Alicante, Spain; 38.299237°, −0.518894°), kept on ice and protected from light until processing. Samples were cleaned and morphologically identified using a Leica MZ6 stereomicroscope (Leica Microsystems, Wetzlar, Germany) and an Olympus BH-2 microscope (Olympus Corporation, Hachioji, Tokyo, Japan) equipped with a Leica DFC camera [70].

For molecular identification, DNA was extracted using a CTAB-based buffer (0.1 M Tris-HCl, 0.05 M EDTA, 1.5 M NaCl, 0.05 M DTT and 3% CTAB) [71].

The cytochrome oxidase I (COI) gene was amplified using primers GazF1 and GazR1 [72]. PCR conditions consisted of an initial denaturation at 94 °C for 4 min, followed by 40 cycles (94 °C for 1 min, 50 °C for 30 s, and 72 °C for 1 min), and a final extension at 72 °C for 7 min. PCR products were visualized on a 1% agarose gel, purified (GeneJET Extraction Kit, ThermoScientific, Waltham, MA, USA) and sequenced by the Sanger method (STAB Vida, Portugal).

Consensus sequences were generated using BioEdit v7.2.5 [73] and compared with the NCBI database using megaBLAST (accessed 10 February 2024).

### 4.4. Extraction of the Sulphated Polysaccharide-Rich Extract from Jania pedunculata var. adhaerens

Lyophilized (CHRIST Alpha 1-2 LDplus, Martin Christ Gefriertrocknungsanlagen GmbH, Osterode am Harz, Germany) and ground algal biomass was treated with 80% ethanol at 80 °C for 4 h to remove pigments, phenols, proteins and salts. This step was repeated until complete pigment removal. The sample was filtered and dried, then dissolved in ultrapure water (1:30, *w*/*v*) and heated at 100 °C for 1 h [73,74]. After filtration and centrifugation (4000× *g*, 10 min, 4 °C), the supernatant was dialyzed for 48 h using a 10 kDa cut-off membrane. Polysaccharides were precipitated by adding two volumes of 95% ethanol and incubating at −20 °C for 48 h [75]. The solution was centrifuged (4000× *g*, 10 min, 4 °C), and the pellet was lyophilized and resuspended in sterile water to obtain a final stock of 10 mg mL^−1^. Extraction yield (%) was calculated as the dry weight of the lyophilized extract relative to the initial dry weight of lyophilized algal biomass × 100.

#### 4.4.1. Characterization of Sulphated Polysaccharide-Rich Extract

The extract was considered a sulphated polysaccharide-rich extract rather than a purified polysaccharide fraction. Therefore, its composition was assessed by determining lipids, soluble and insoluble carbohydrates, proteins, mineral elements (Ca, Mg, K, Na and S), total sulphated polysaccharides and sulphur content. Lipids were extracted by Soxhlet extraction using petroleum ether (40–60 °C), as previously described [76]. Carbohydrates were determined using the anthrone method, distinguishing soluble and insoluble fractions [77]. Sulphated polysaccharides were quantified using a turbidimetric method [78]. Protein content was determined by the Bradford assay [79]. Elemental composition (Ca, Mg, Na and K) was analyzed by ICP-OES (Perkin Elmer 7300DV, PerkinElmer, Shelton, CT, USA). FTIR analysis was performed using a JASCO FT/IR-4700 spectrophotometer (JASCO Corporation, Tokyo, Japan) (500–4000 cm^−1^, ATR mode).

#### 4.4.2. Monosaccharide Composition

Polysaccharides (10 mg) were hydrolyzed with 6 M HCl at 110 °C for 6 h. After solvent evaporation, samples were resuspended in ultrapure water (100 µg mL^−1^) and filtered (0.22 µm). Monosaccharides were analyzed by anion-exchange chromatography with pulsed amperometric detection.

### 4.5. Antifungal Activity Assays

Antifungal activity was evaluated by growth kinetics in liquid medium. Yeasts were grown on Sabouraud medium (CondaLab, Ref. 1134, Madrid, Spain) for 24 h at 36 ± 1 °C. A single colony was then transferred to YPD medium (yeast extract, peptone, dextrose) and incubated under the same conditions. Cell suspensions were adjusted to OD_490_ < 0.1 [80]. All treatments were prepared in 50% YPD medium. Polysaccharides were tested at 1, 2, 3 and 5 mg mL^−1^, and chitosan at 2.5, 5, 10, 15 and 20 µg mL^−1^. Cultures were incubated for 24 h at 36 ± 1 °C, and optical density was measured hourly at 490 nm using an Infinite F Nano multimode microplate reader (Tecan Trading AG, Zurich, Switzerland). All assays were performed in three independent biological experiments, with three technical replicates per treatment in each experiment. Minimum inhibitory concentration (MIC) was estimated from growth kinetics as the lowest concentration showing no detectable increase in OD_490_ during the assay, together with the statistical analysis of the growth rate parameter (*r*) [45,81].

### 4.6. Statistical Analysis

Growth curves were analyzed by fitting an exponential model: N(t) = N_0_e^rt^, where r is the growth rate parameter. The model was fitted using the Levenberg–Marquardt algorithm (minpack.lm package, R package v1.2-4, 2023 [82]). Differences between treatments were analyzed by one-way ANOVA. Growth inhibition (%) was calculated at 20 h relative to the control. Normality and homoscedasticity were tested using the Shapiro–Wilk and Levene tests, respectively, and Tukey HSD was used for post hoc comparisons. K-means clustering was used to group species based on sensitivity, and the optimal number of clusters was determined using silhouette analysis. Principal component analysis was used to visualize the K-means clusters in two-dimensional space. All analyses were performed in R v4.3.3 [83].

A schematic representation of the statistical workflow used in this study is provided in Figure S14.

## 5. Conclusions

Chitosan exhibited antifungal activity across a broad panel of clinically relevant yeasts, while the sulphated polysaccharide-rich extract showed moderate antifungal activity against the four selected species evaluated. However, antifungal efficacy strongly depended on both antifungal concentration and target species, highlighting marked interspecific variability in susceptibility. Chitosan showed higher potency at low concentrations, whereas the algal sulphated polysaccharide-rich extract displayed a moderate but consistent inhibitory effect.

The contrasting responses observed between treatments suggest distinct mechanisms of action, which may offer complementary antifungal strategies. In particular, the sensitivity of multidrug-resistant species such as *Candida auris* to chitosan supports further evaluation of chitosan against *Candida auris*.

Overall, these findings support marine-derived biopolymers as promising candidates for antifungal applications and encourage further studies to evaluate their mechanisms and potential combined use.

## Figures and Tables

**Table 1:** Minimum inhibitory concentration (MIC) and maximum growth inhibition values of an aqueous solution of chitosan and the sulphated polysaccharide-rich extract (PS) extracted from *Jania pedunculata* var. *adhaerens* against different yeast species. MIC values are expressed as µg mL^−1^ for chitosan and mg mL^−1^ for the sulphated polysaccharide-rich extract. Maximum growth inhibition refers to the highest percentage reduction in OD_490_ at 20 h relative to the untreated control. Values are presented as mean ± standard deviation. † No detectable increase in OD_490_ was observed during the assay, indicating apparent growth suppression under the tested conditions. “-” indicates species that were not evaluated with the sulphated polysaccharide-rich extract.

Species	MIC Chitosan (µg mL^−1^)	MIC PS (mg mL^−1^)	Max Inhibition Chitosan (%)	Max Inhibition PS (%)
*C. albicans*	>20	>5	11.5 ± 3.21	59.8 ± 2.69
*C. auris*	20	>5	78.2 ± 1.26 †	55.02 ± 7.01
*C. glabrata*	20	-	69.8 ± 0.74 †	-
*C. guilliermondii*	>20	-	72.2 ± 0.28	-
*C. parapsilosis*	15	-	82.6 ± 0.32 †	-
*Cr. bacillisporus*	10	>5	76.7 ± 0.87 †	30.39 ± 3.55
*Cr. deneoformans*	15	-	87.7 ± 1.04 †	-
*Cr. deuterogattii*	15	>5	74.8 ± 3.09 †	60.88 ± 1.94
*Cr. tetragattii*	20	-	85.1 ± 0.35 †	-
*Cl. lusitaniae*	20	-	87.0 ± 0.26 †	-
*N. albida*	20	-	81.2 ± 0.55 †	-
*T. cutaneum*	>20	-	69.4 ± 3.59	-

## Data Availability

The original contributions presented in this study are included in the article and Supplementary Materials. Further inquiries can be directed to the corresponding authors.

## Supplementary Materials

The following supporting information can be downloaded at https://www.mdpi.com/article/10.3390/md24090296/s1, Figure S1: Morphological characterization of *Jania pedunculata* var. *adhaerens*; Figure S2: Growth kinetics of *C. albicans*, *C. auris*, *Cr. bacillisporus* and *Cr. deuterogattii* exposed to different concentrations of chitosan; Figure S3: Growth kinetics of *C. parapsilosis* and *Cryptococcus* spp. exposed to different concentrations of chitosan; Figure S4: Growth kinetics of *Cr. tetragattii*, *Cl. lusitaniae*, *N. albida* and *T. cutaneum* exposed to different concentrations of chitosan; Table S1: Growth rate parameter (r) of yeast species exposed to different concentrations of chitosan; Table S2: One-way ANOVA results for the growth rate parameter (r) of yeast species exposed to different concentrations of chitosan; Figure S5: Effect of chitosan on the growth parameter (r) of *C. albicans*, *C. auris*, *C. glabrata* and *C. guilliermondii*; Figure S6: Effect of chitosan on the growth parameter (r) of *C. parapsilosis*, *Cr. bacillisporus*, *Cr. deneoformans* and *Cr. deuterogattii*; Figure S7: Effect of chitosan on the growth parameter (r) of *Cr. tetragattii*, *Cl. lusitaniae*, *N. albida* and *T. cutaneum*; Table S3: One-way ANOVA results for growth inhibition of yeast species exposed to different concentrations of chitosan; Figure S8: Effect of chitosan on growth inhibition (%) of *C. albicans*, *C. auris*, *C. glabrata* and *C. guilliermondii*; Figure S9: Effect of chitosan on growth inhibition (%) of *C. parapsilosis*, *Cr. bacillisporus*, *Cr. deneoformans* and *Cr. deuterogattii*; Figure S10: Effect of chitosan on growth inhibition (%) of *Cr. tetragattii*, *Cl. lusitaniae*, *N. albida* and *T. cutaneum*; Figure S11: Growth kinetics of *C. albicans*, *C. auris*, *Cr. bacillisporus* and *Cr. deuterogattii* exposed to different concentrations of seaweed polysaccharides; Table S4: Growth rate parameter (r) of yeast species exposed to different concentrations of seaweed polysaccharides; Table S5: One-way ANOVA results for the growth rate parameter (r) of yeast species exposed to different concentrations of seaweed polysaccharides; Figure S12: Effect of seaweed polysaccharides on the growth parameter (r) of *C. albicans*, *C. auris*, *Cr. bacillisporus* and *Cr. deuterogattii*; Table S6: One-way ANOVA results for growth inhibition of yeast species exposed to different concentrations of seaweed polysaccharides; Figure S13: Effect of seaweed polysaccharides on growth inhibition (%) of *C. albicans*, *C. auris*, *Cr. bacillisporus* and *Cr. deuterogattii*; Figure S14: Schematic representation of the statistical workflow applied to evaluate the antifungal activity of chitosan and sulfated polysaccharides against yeast species.
