# Supplementary material for: Species-Dependent Antifungal Profiles of Chitosan and Sulphated Polysaccharide-Rich Extract from Jania pedunculata var. adhaerens
Source: Mar Drugs. 2026 Aug 24;24(9):296. doi: 10.3390/md24090296 (PMC13608624; doi:10.3390/md24090296)
Supplement: Supplementary file 1 [file marinedrugs-24-00296-s001.zip › marinedrugs-4482510-supplementary.pdf]

Supplementary material

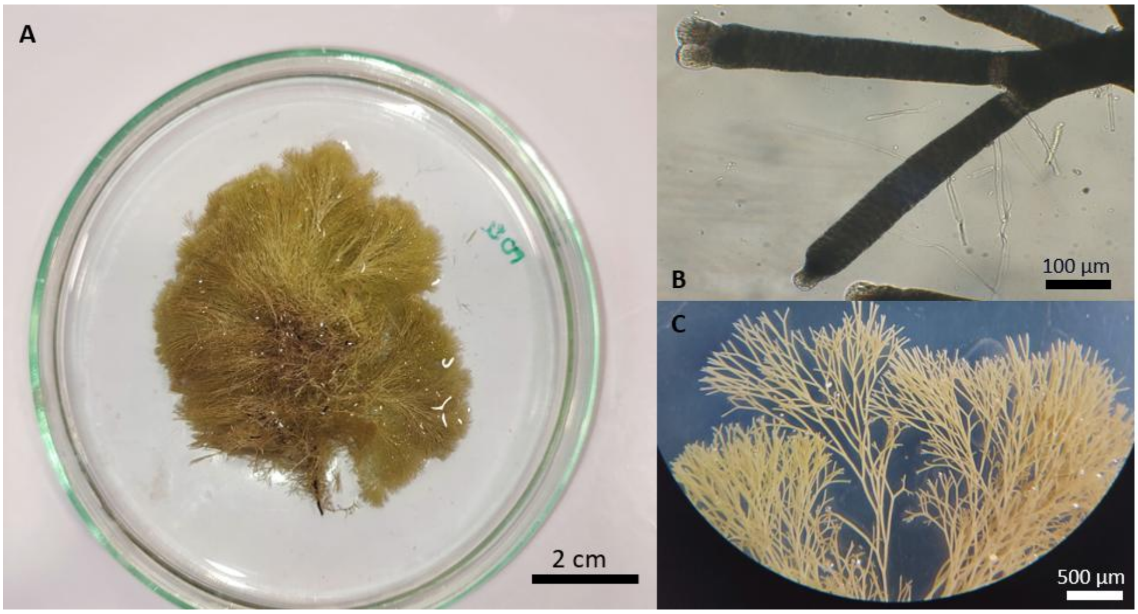

Figure S1. Morphological characterization of *Jania pedunculata* var. *adhaerens*. (A) General appearance of the alga, showing a dichotomous thallus with a pale pink-lilac coloration and an approximate length of 5 cm. (B) Detail of the calcified intergenicula, which presented diameters ranging from 80 to 120 µm and lengths approximately 5–6 times greater than their width. (C) Representative branching pattern of the thallus, with dichotomy angles greater than 30°.

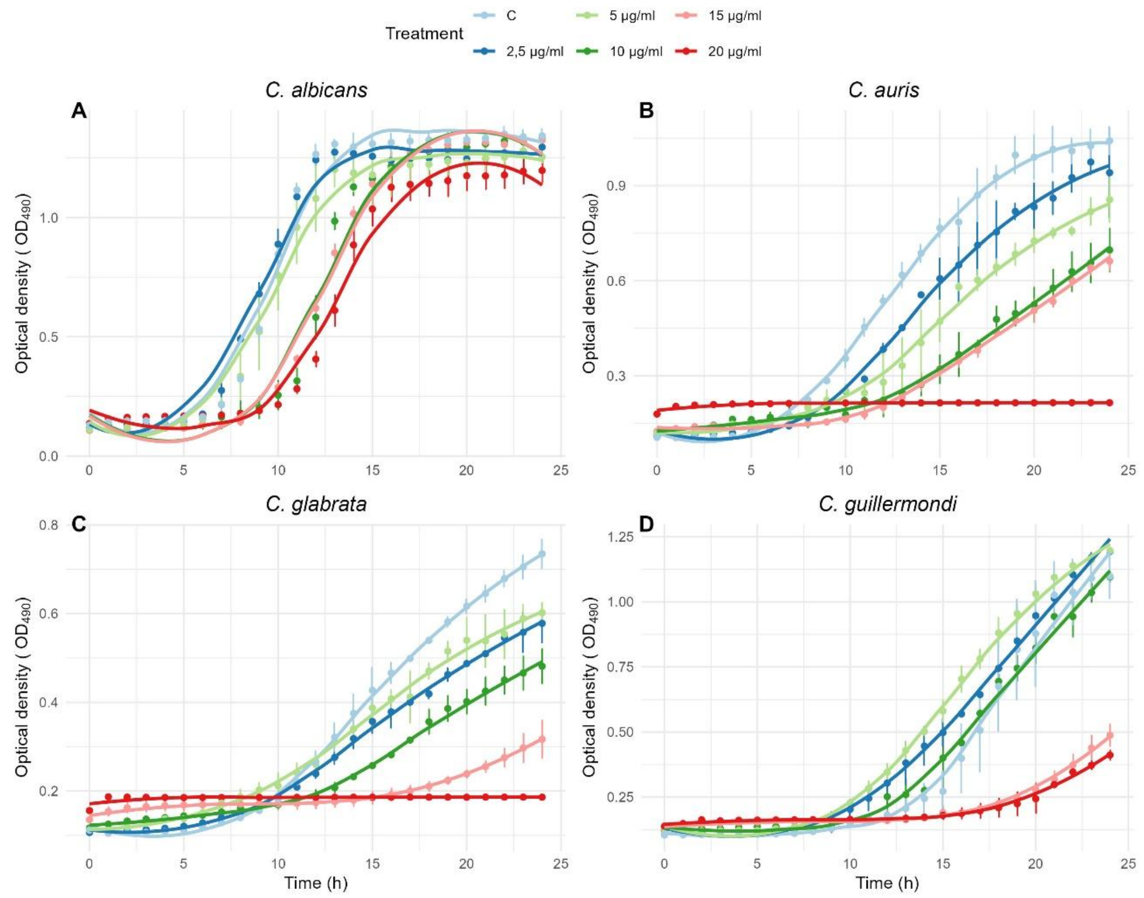

Figure S2: Growth kinetics of *C. albicans* (A), *C. auris* (B), *Cr. bacillisporus* (C) and *Cr. deuterogatti* (D) exposed to different concentrations of chitosan over 24 h. Growth was monitored by OD490 measurements. Treatment indicates the chitosan concentrations applied. C: control. Values represent mean ± standard deviation.

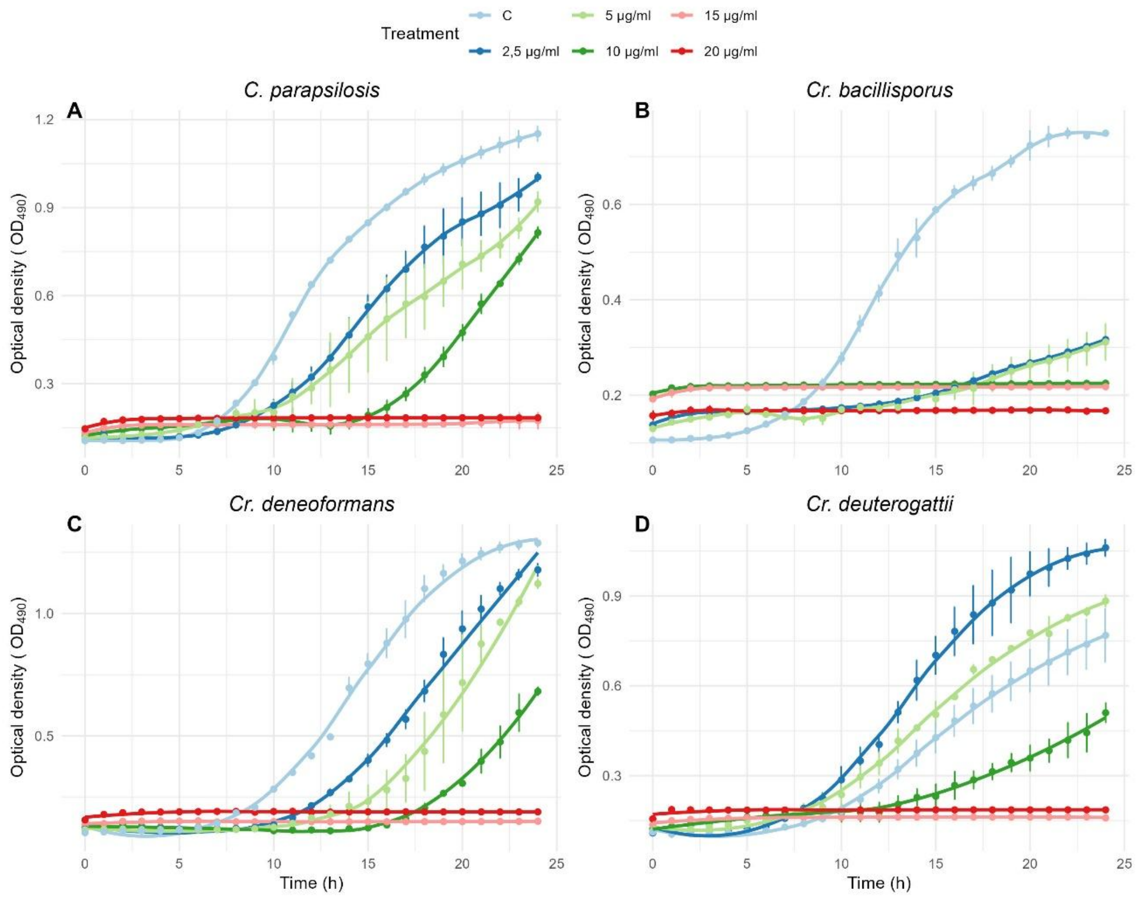

Figure S3. Growth kinetics of *C. parapsilosis* (A) and *Cryptococcus* spp. including *Cr. bacillisporus* (B), *Cr. deneoformans* (C) and *Cr. deuterogatti* (D) exposed to different concentrations of chitosan over 24 h. Growth was monitored by OD490 measurements. Treatment indicates the chitosan concentrations applied. C: control. Values represent mean ± standard deviation.

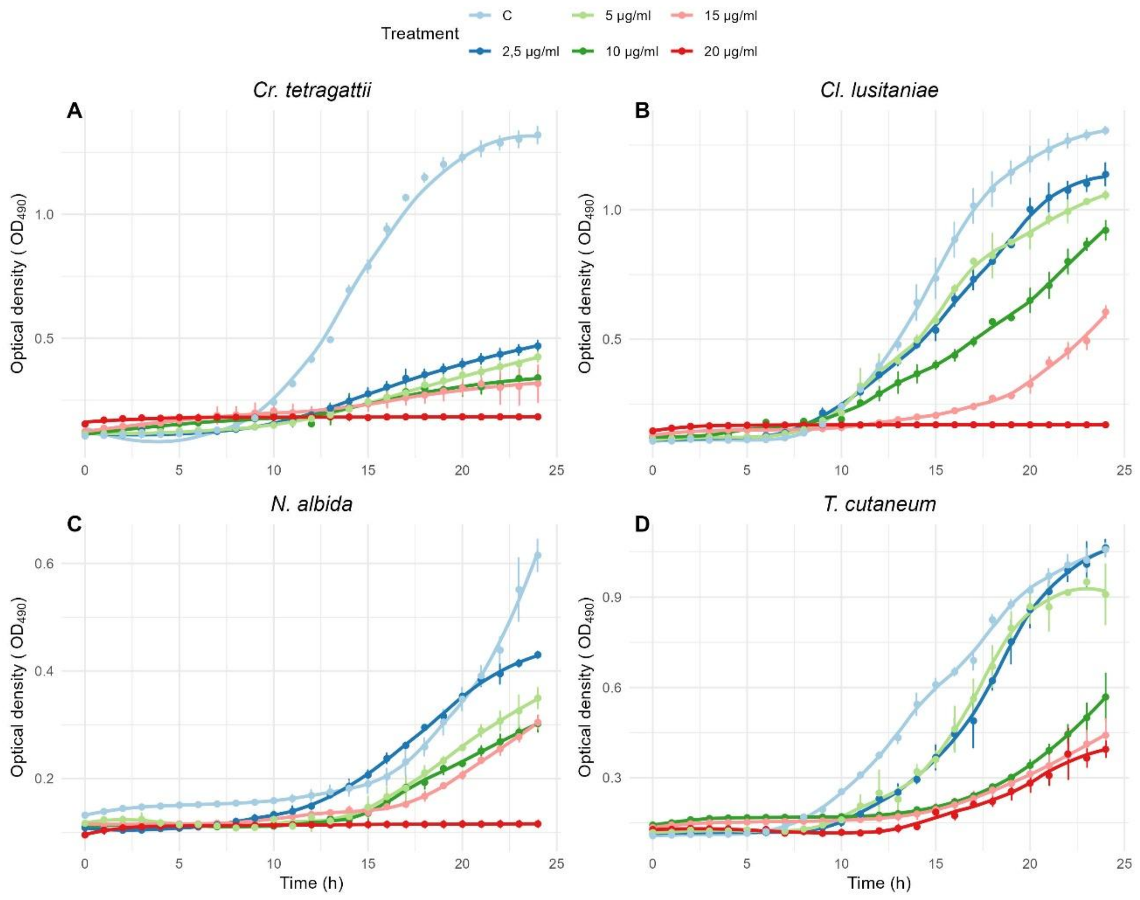

Figure S4. Growth kinetics of *Cr. tetragattii* (A), *Cl. lusitaniae* (B), *N. albida* (C) and *T. cutaneum* (D) exposed to different concentrations of chitosan over 24 h. Growth was monitored by OD490 measurements. Treatment indicates the chitosan concentrations applied. C: control. Values represent mean ± standard deviation.

Table S1. Growth rate parameter (r) obtained from the exponential growth model fitted for each yeast species exposed to different concentrations of chitosan. Estimation values, standard errors, t values and p values are shown for each treatment condition.

Species	Treatment	Estimate	Standard error	T value	p.value
C. albicans					
	C	0.186	0.003	58.04	**<0.001**
	2.5 µg/ml	0.185	0.003	56.93	**<0.001**
	5 µg/ml	0.178	0.003	55.72	**<0.001**
	10 µg/ml	0.159	0.003	40.23	**<0.001**
	15 µg/ml	0.156	0.003	45.64	**<0.001**
	20 µg/ml	0.141	0.004	34.8	**<0.001**
C. auris					
	C	0.429	0.020	20.82	**<0.001**
	2.5 µg/ml	0.363	0.019	18.84	**<0.001**
	5 µg/ml	0.394	0.044	8.85	**<0.001**
	10 µg/ml	0.148	0.032	4.562	**<0.001**
	15 µg/ml	0.204	0.023	8.61	**<0.001**

	20 µg/ml	0.050	0.006	7.62	**<0.001**
C. glabrata					
	C	0.114	0.004	28.32	**<0.001**
	2.5 µg/ml	0.094	0.002	32.75	**<0.001**
	5 µg/ml	0.090	0.003	29.47	**<0.001**
	10 µg/ml	0.081	0.002	29.10	**<0.001**
	15 µg/ml	0.022	0.002	9.08	**<0.001**
	20 µg/ml	0.0002	0.0001	2.70	**<0.001**
C. guillermondii					
	C	0.125	0.027	4.530	**<0.001**
	2.5 µg/ml	0.175	0.023	7.631	**<0.001**
	5 µg/ml	0.181	0.010	71.008	**<0.001**
	10 µg/ml	0.189	0.037	5.019	**<0.001**
	15 µg/ml	0.036	0.009	3.943	**0.001**
	20 µg/ml	0.017	0.006	2.71	**0.015**
C. parapsilosis					
	C	0.173	0.009	18.9	**<0.001**
	2.5 µg/ml	0.177	0.007	23.03	**<0.001**
	5 µg/ml	0.130	0.017	7.49	**<0.001**
	10 µg/ml	0.003	0.009	0.417	0.63
	15 µg/ml	0.001	0.0001	5.90	**<0.001**
Cr. bacillisporus					
	C	0.158	0.005	29.24	**<0.001**
	2.5 µg/ml	0.020	0.001	15.85	**<0.001**
	5 µg/ml	0.023	0.004	5.037	**<0.001**
	10 µg/ml	0.001	0.001	1.318	0.197
	15 µg/ml	0.0004	0.0006	0.775	0.444
	20 µg/ml	0.0004	0.0004	1.016	0.317
Cr. deneoformans					
	C	0.168	0.005	29.79	**<0.001**
	2.5 µg/ml	0.176	0.004	40.21	**<0.001**
	5 µg/ml	0.177	0.155	11.409	**<0.001**
	10 µg/ml	0.076	0.008	9.475	**<0.001**
	15 µg/ml	-0.006	0.002	-0.322	0.749
	20 µg/ml	-0.0006	0.0005	-1.23	0.226
Cr. deuterogatti					
	C	0.166	0.014	11.37	**<0.001**
	2.5 µg/ml	0.180	0.014	12.835	**<0.001**
	5 µg/ml	0.142	0.013	10.44	**<0.001**
	10 µg/ml	0.065	0.021	3.098	**<0.01**
	15 µg/ml	0.0008	0.007	0.117	0.908
	20 µg/ml	0.0004	0.0005	0.863	0.401
Cr. tetragattii					
	C	0.131	0.008	16.003	**<0.001**
	2.5 µg/ml	0.091	0.004	18.33	**<0.001**
	5 µg/ml	0.087	0.004	18.09	**<0.001**
	10 µg/ml	0.064	0.007	8.375	**<0.001**
	15 µg/ml	0.045	0.006	6.662	**<0.001**
Cl. lusitaniae					
	C	0.177	0.001	152.7	**<0.001**
	2.5 µg/ml	0.159	0.0007	211.3	**<0.001**
	5 µg/ml	0.163	0.0009	181.2	**<0.001**

	10 µg/ml	0.137	0.001	103.7	**<0.001**
	15 µg/ml	0.096	0.001	70.26	**<0.001**
	20 µg/ml	0.084	0.002	29.39	0.226
N. albida					
	C	0.100	0.001	95.67	**<0.001**
	2.5 µg/ml	0.093	0.006	149.7	**<0.001**
	5 µg/ml	0.081	0.0006	122.8	**<0.001**
	10 µg/ml	0.075	0.0005	146.8	**<0.001**
	15 µg/ml	0.072	0.0006	116.2	**<0.001**
	20 µg/ml	0.040	0.001	35.03	**<0.001**
T. cutaneum					
	C	0.121	0.004	26.27	**<0.001**
	2.5 µg/ml	0.172	0.005	30.625	**<0.001**
	5 µg/ml	0.170	0.006	26.00	**<0.001**
	10 µg/ml	0.066	0.003	17.16	**<0.001**
	15 µg/ml	0.066	0.004	14.59	**<0.001**
	20 µg/ml	0.088	0.005	17.07	**<0.001**

Table S2. One-way ANOVA results for the growth rate parameter (r) of the different yeast species exposed to different concentrations of chitosan. Df: degrees of freedom. Sum Sq: sum of squares. Mean Sq: mean squares. F value: F statistic value.

Species	Df	Sum Sq	Mean Sq	F value	p.value
C. albicans	5	0.351	0.070	123.4	**<0.001**
	12	0.006	0.0005		
C. auris	5	0.066	0.013	23.28	**<0.001**
	12	0.006	0.0005		
C. glabrata	5	0.058	0.011	128.7	**<0.001**
	12	0.001	0.00009		
C. guillermondii	5	0.086	0.017	19.93	**<0.001**
	12	0.104	0.0008		
C. parapsilosis	5	0.114	0.022	117.4	**<0.001**
	12	0.002	0.0001		
Cr. bacillisporus	5	0.057	0.011	519.8	**<0.001**
	12	0.0006	0.00002		
Cr. deneoformans	5	0.132	0.026	91.52	**<0.001**
	12	0.003	0.0002		
C. deuterogatti	5	0.100	0.020	45.28	**<0.001**
	12	0.005	0.0004		
C. tetragattii	5	0.029	0.005	44.45	**<0.001**
	12	0.001	0.001		
Cl. lusitaniae	5	0.020	0.004	1033	**<0.001**
	12	0.00004	0.00004		
N. albida	5	0.005	0.001	206.4	**<0.001**
	12	0.00006	0.000005		
T. cutaneum	5	0.014	<0.001	153	**<0.001**
	12	0.0002	<0.001		

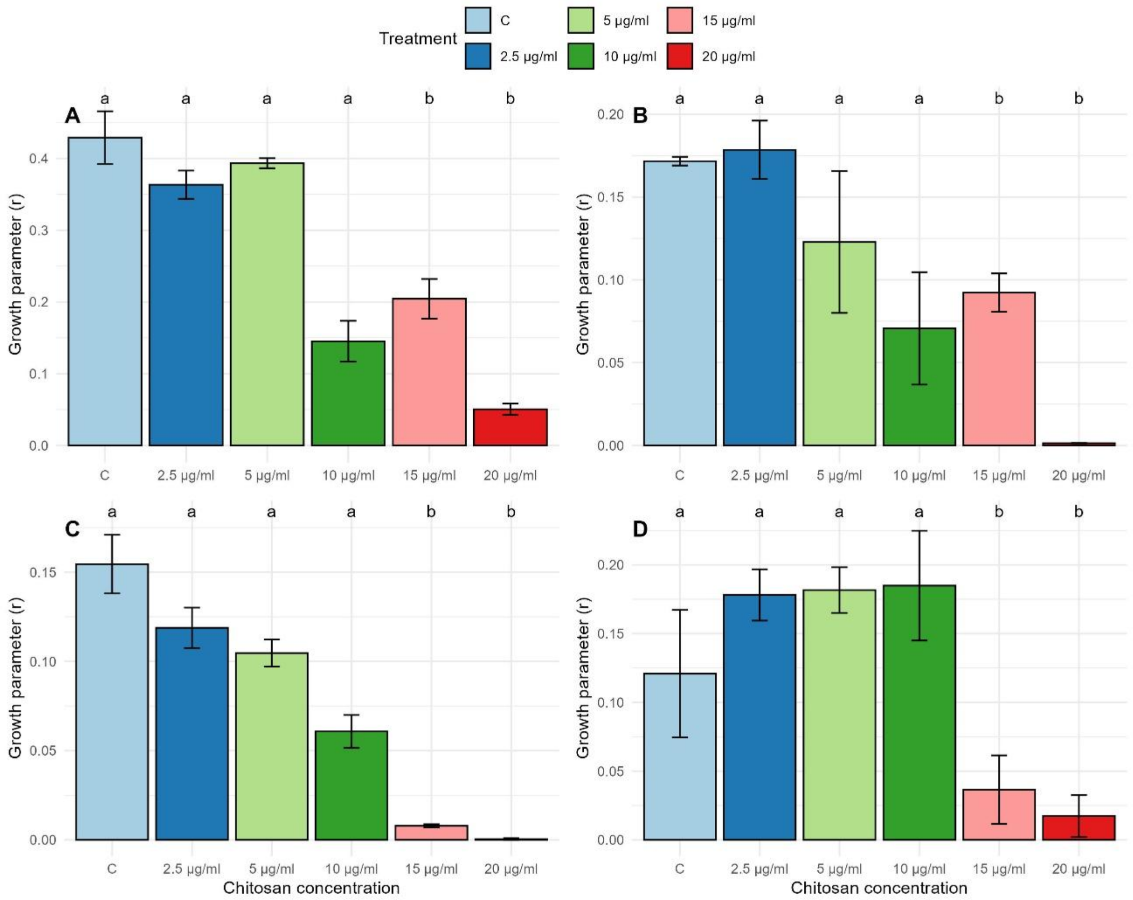

Figure S5. Effect of chitosan on the growth parameter (r) of A) *C. albicans*, B) *C. auris*, C) *C. glabrata*, and D) *C. guilliermondii* at different concentrations (2.5, 5, 10, 15, and 20 µg mL^{-1}). Bars represent the mean ± standard deviation. Different letters indicate significant differences among treatments according to Tukey's post hoc test ($p < 0.05$).

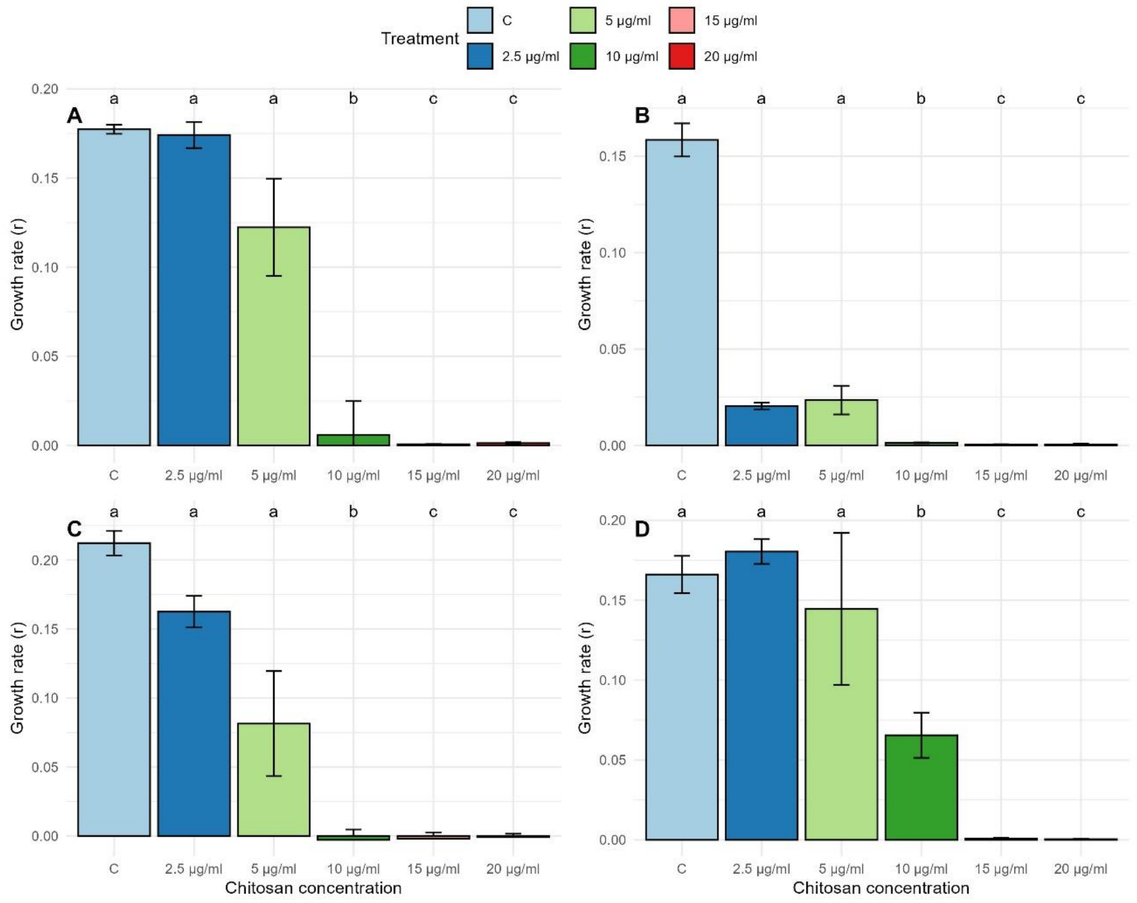

Figure S6. Effect of chitosan on the growth parameter (r) of A) *C. parapsilosis*, B) *Cr. bacillisporus*, C) *Cr. deneoformans* and D) *Cr. deuterogatti* at different concentrations (2.5, 5, 10, 15, and 20 µg mL⁻¹). Bars represent the mean ± standard deviation. Different letters indicate significant differences among treatments according to Tukey's post hoc test ($p < 0.05$).

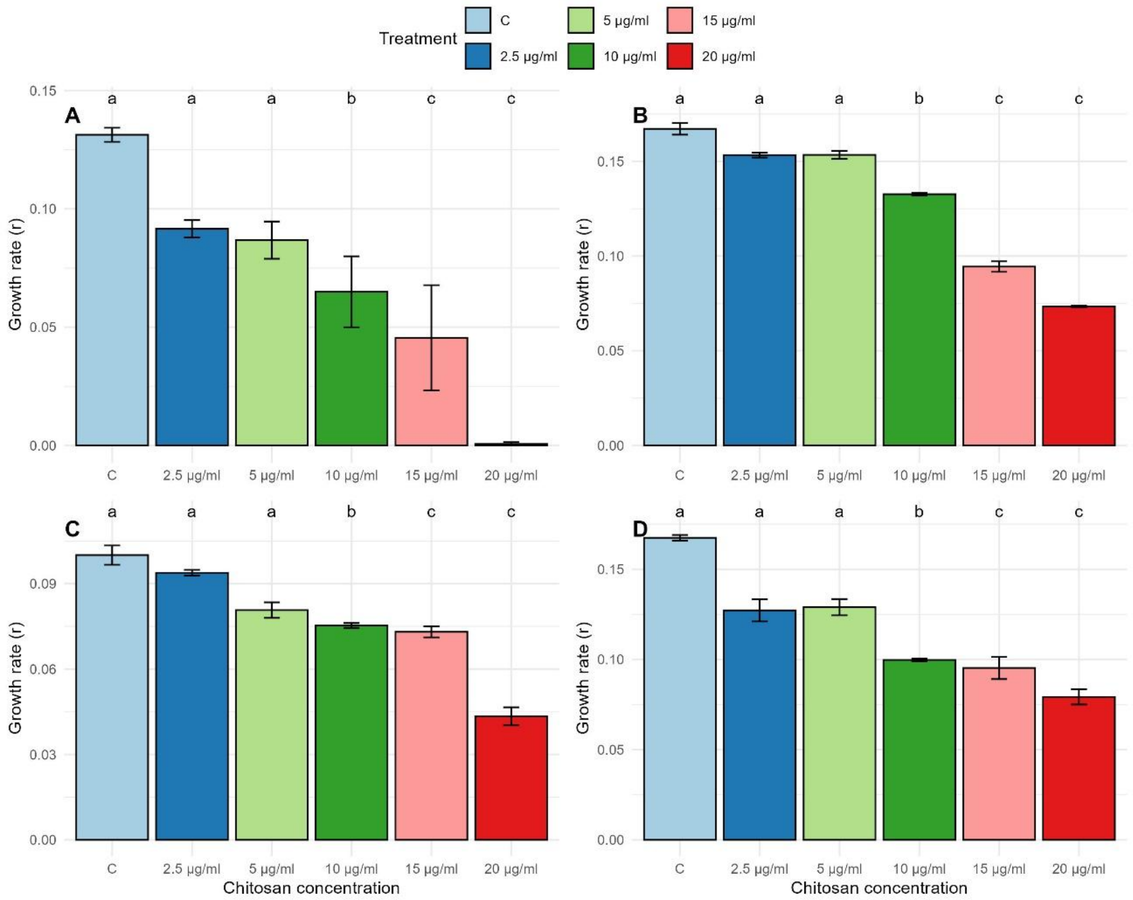

Figure S7. Effect of chitosan on the growth parameter (r) of A) *Cr. tetragattii*, B) *Cl. lusitaniae*, C) *N. albida* y D) *T. cutaneum* at different concentrations (2.5, 5, 10, 15, and 20 µg mL^{-1}). Bars represent the mean ± standard deviation. Different letters indicate significant differences among treatments according to Tukey's post hoc test ($p < 0.05$).

Table S3. One-way ANOVA results for the growth inhibition of the different yeast species exposed to different concentrations of chitosan. Df: degrees of freedom. Sum Sq: sum of squares. Mean Sq: mean squares. F value: F statistic value.

Species	Df	Sum Sq	Mean Sq	F value	p.value
C. albicans	4	211	52.75	4.12	**<0.001**
	10	128	12.80		
C. auris	4	6925	1731	75.22	**<0.001**
	10	230	23		
C. glabrata	4	7465	1866.3	117.8	**<0.001**
	10	158	15.8		
C. guillermondii	4	23950	5987	14.48	**<0.001**
	10	4135	414		
C. parapsilosis	4	10118	2529.5	83.08	**<0.001**
	10	304	30.4		
Cr. bacillisporus	4	372.6	93.16	31.57	**<0.001**
	10	29.5	2.95		
Cr. deneoformans	4	9954	2488.4	34.01	**<0.001**
	10	732	73.2		

C. deuterogatti	4	38309	9577	82.03	**<0.001**
	10	1168	117		
C. tetragattii	4	503.0	125.75	38.27	**<0.001**
	10	32.9	3.9		
Cl. lusitaniae	4	11028	2765.9	428.2	**<0.001**
	10	64	6.4		
N. albida	4	4243	1061	96.43	**<0.001**
	10	110	11		
T. cutaneum	4	12933	3233	131.9	**<0.001**
	10	245	25		

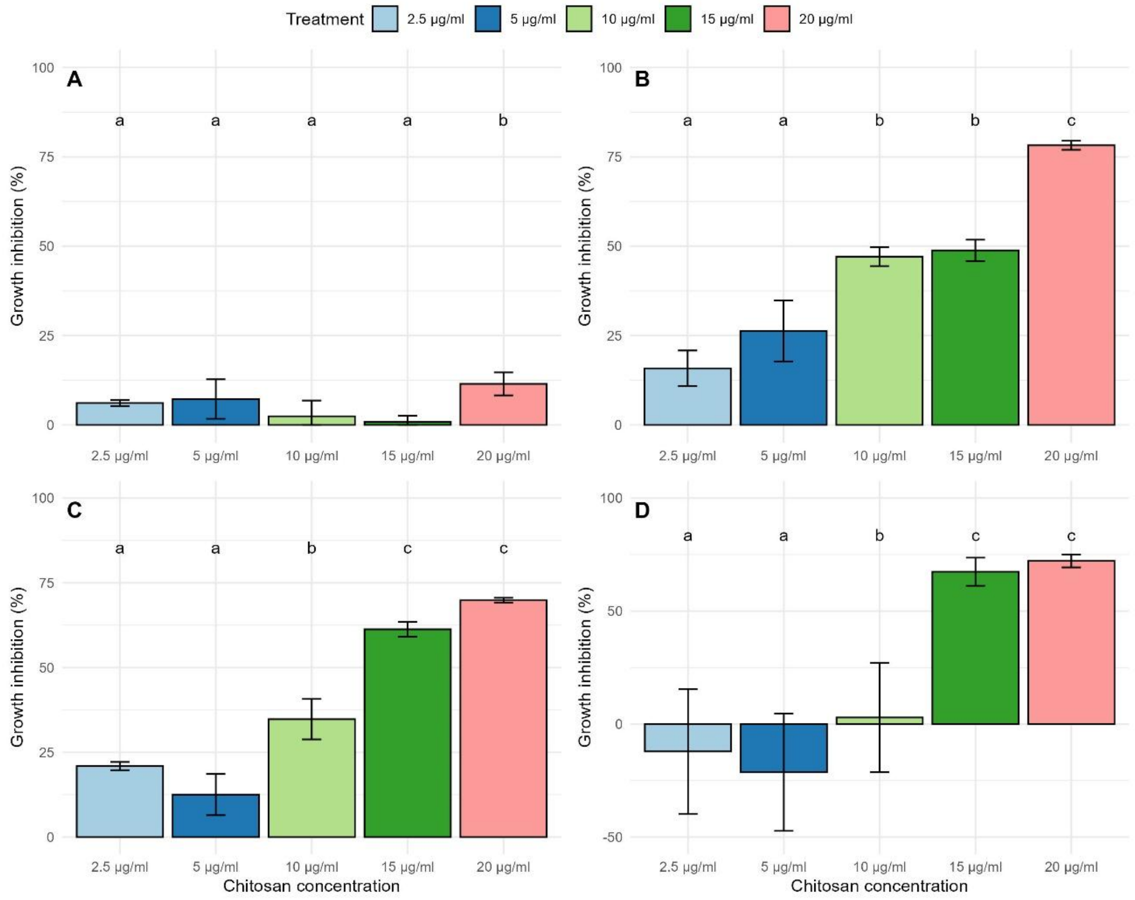

Figure S8. Effect of chitosan on growth inhibition (%) of (A) *C. albicans*, (B) *C. auris*, (C) *C. glabrata*, and (D) *C. guilliermondii* at different concentrations (2.5, 5, 10, 15, and 20 µg mL^{-1}). Bars represent the mean ± standard deviation. Different letters indicate significant differences among treatments according to Tukey's post hoc test ($p < 0.05$).

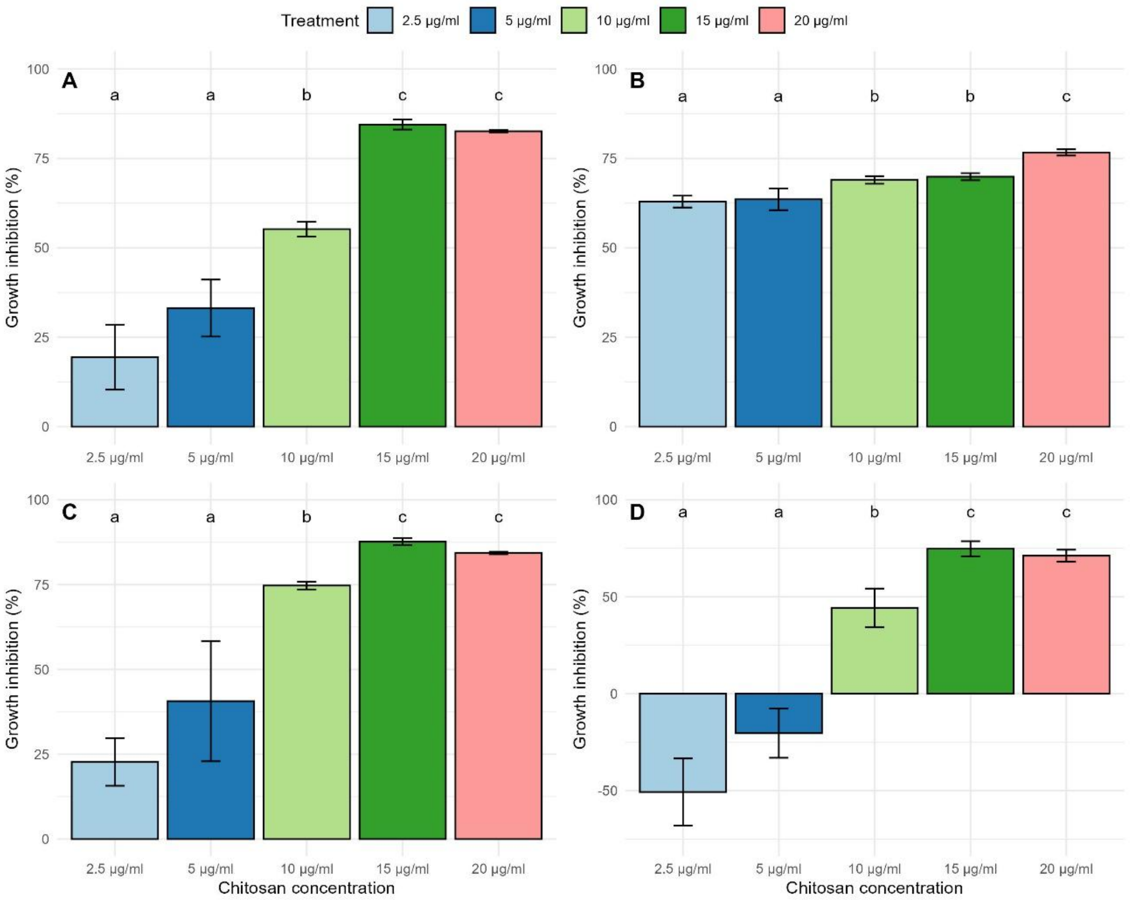

Figure S9. Effect of chitosan on growth inhibition (%) of A) *C. parapsilosis*, B) *Cr. bacillisporus*, C) *Cr. deneoformans* and D) *Cr. deuterogatti* at different concentrations (2.5, 5, 10, 15, and 20 µg mL^{-1}). Bars represent the mean ± standard deviation. Different letters indicate significant differences among treatments according to Tukey's post hoc test ($p < 0.05$).

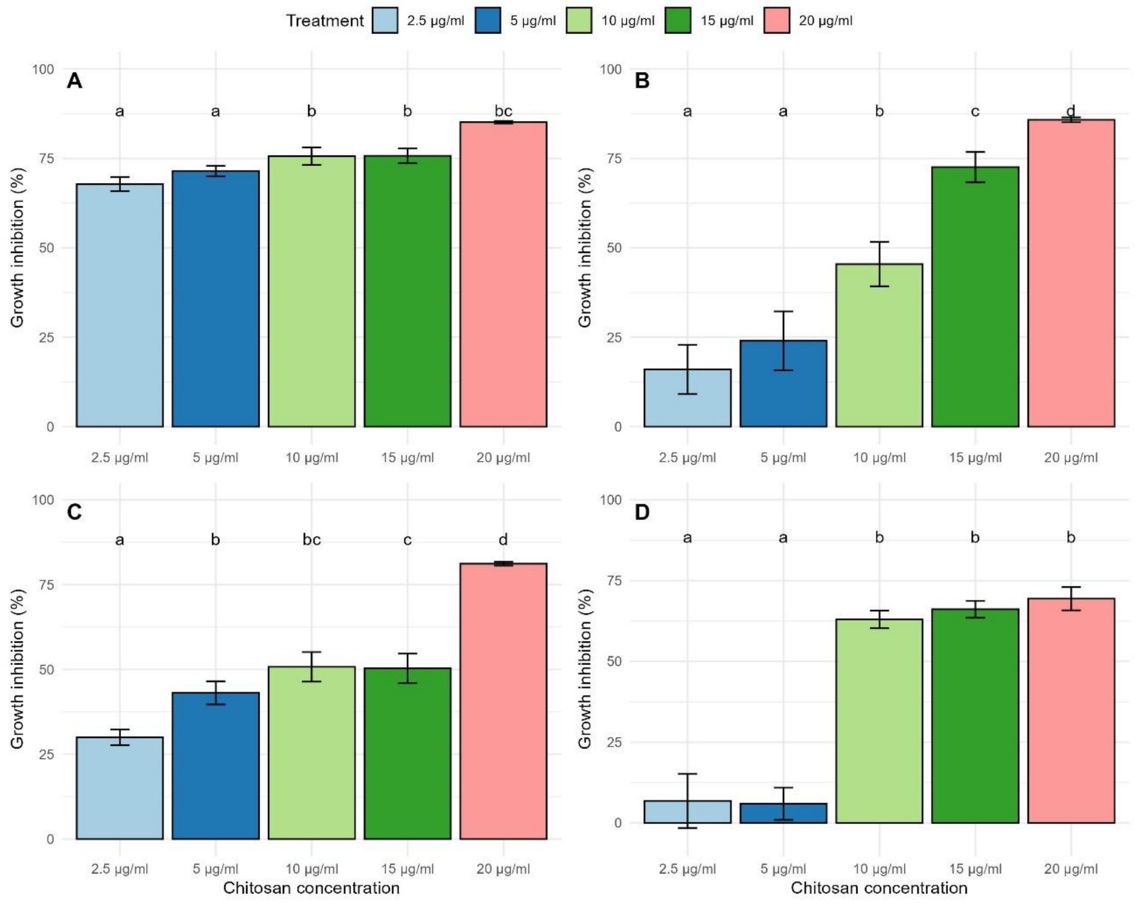

Figure S10. Effect of chitosan on growth inhibition (%) of A) *Cr. tetragattii*, B) *Cl. lusitaniae*, C) *N. albida* and D) *T. cutaneum* at different concentrations (2.5, 5, 10, 15, and 20 µg mL^{-1}). Bars represent the mean ± standard deviation. Different letters indicate significant differences among treatments according to Tukey's post hoc test ($p < 0.05$).

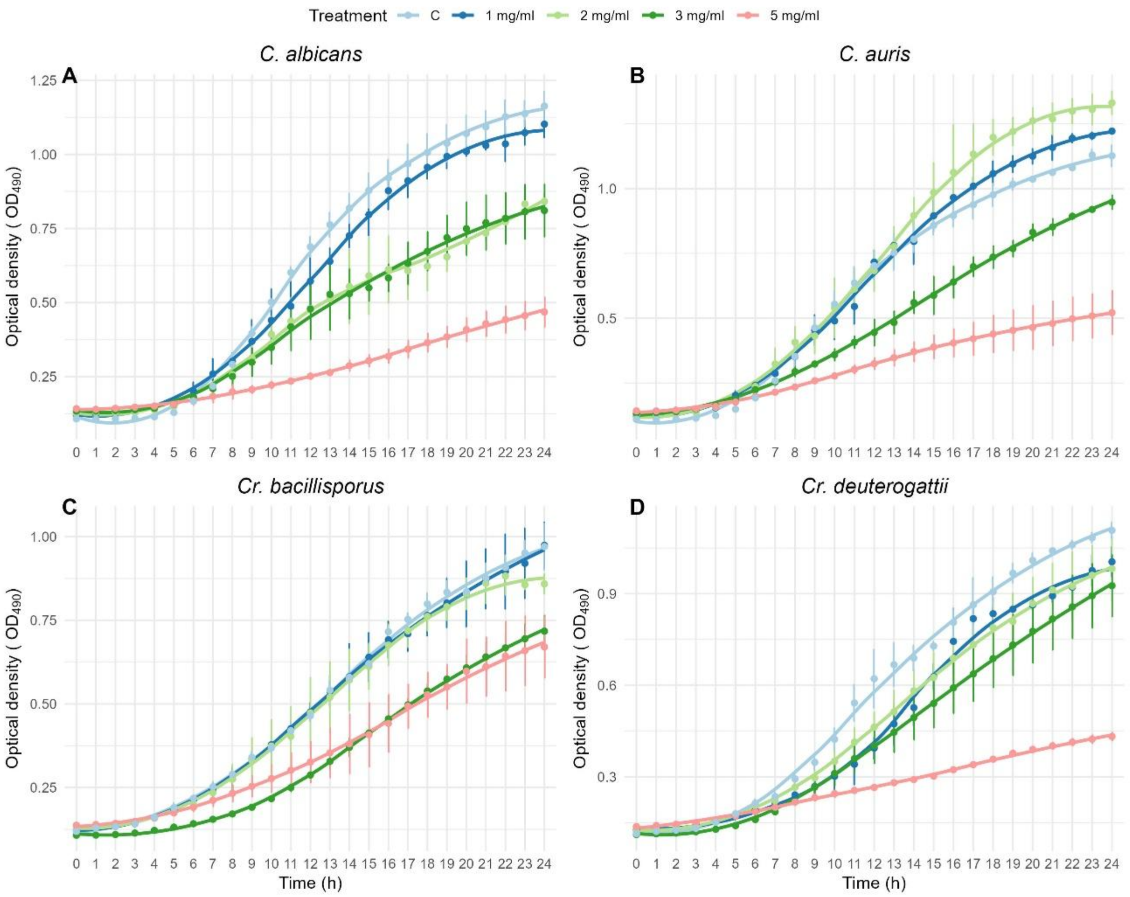

Figure S11. Growth kinetics of *C. albicans* (A), *C. auris* (B), *Cr. bacillisporus* (C) and *Cr. deuterogattii* (D) exposed to different concentrations of seaweed polysaccharides over 24 h. Growth was monitored by OD490 measurements. Treatment indicates the polysaccharides concentrations applied. C: control. Values represent mean ± standard deviation.

Table S4. Growth rate parameter (r) obtained from the exponential growth model fitted for each yeast species exposed to different concentrations of seaweed polysaccharides. Estimation values, standard errors, t values and p values are shown for each treatment condition.

Species	Treatment	Estimate	Standard error	T value	p.value
C. albicans					
	C	0.194	0.001	121.4	**<0.001**
	1 $mg\ mL^{-1}$	0.184	0.002	90.25	**<0.001**
	2 $mg\ mL^{-1}$	0.169	0.002	64.07	**<0.001**
	3 $mg\ mL^{-1}$	0.167	0.002	64.75	**<0.001**
	5 $mg\ mL^{-1}$	0.125	0.002	44.59	**<0.001**
C. auris					
	C	0.203	0.002	89.02	**<0.001**
	1 $mg\ mL^{-1}$	0.202	0.001	103.6	**<0.001**
	2 $mg\ mL^{-1}$	0.203	0.002	76.37	**<0.001**
	3 $mg\ mL^{-1}$	0.171	0.002	67.15	**<0.001**
	5 $mg\ mL^{-1}$	0.148	0.002	52.8	**<0.001**
Cr. bacillisporus					
	C	0.116	0.0005	195.1	**<0.001**
	1 $mg\ mL^{-1}$	0.116	0.001	106.5	**<0.001**
	2 $mg\ mL^{-1}$	0.114	0.001	67.21	**<0.001**

	3 mg mL^{-1}	0.084	0.001	56.25	**<0.001**
	5 mg mL^{-1}	0.089	0.001	45.8	**<0.001**
Cr. deuterogatti					
	C	0.172	0.002	65.71	**<0.001**
	1 mg mL^{-1}	0.158	0.001	102.5	**<0.001**
	2 mg mL^{-1}	0.160	0.002	78.38	**<0.001**
	3 mg mL^{-1}	0.151	0.002	71.38	**<0.001**
	5 mg mL^{-1}	0.118	0.002	49.87	**<0.001**

Table S5. One-way ANOVA results for the growth rate parameter (r) of the different yeast species exposed to different concentrations of seaweed polysaccharides. Df: degrees of freedom. Sum Sq: sum of squares. Mean Sq: mean squares. F value: F statistic value.

Species	Df	Sum Sq	Mean Sq	F value	p.value
C. albicans	4	0,008	0,002	23,71	**<0,001**
	10	0,0009	0,00009		
C. auris	4	0,007	0,001	39,99	**<0,001**
	10	0,0004	0,00004		
C. bacillisporus	4	0,001	<0.001	18,6	**<0,001**
	10	0,0002	<0,001		
Cr. deuterogatti	4	0,004	0,001	51,08	**<0,001**
	10	0,0002	0,00002		

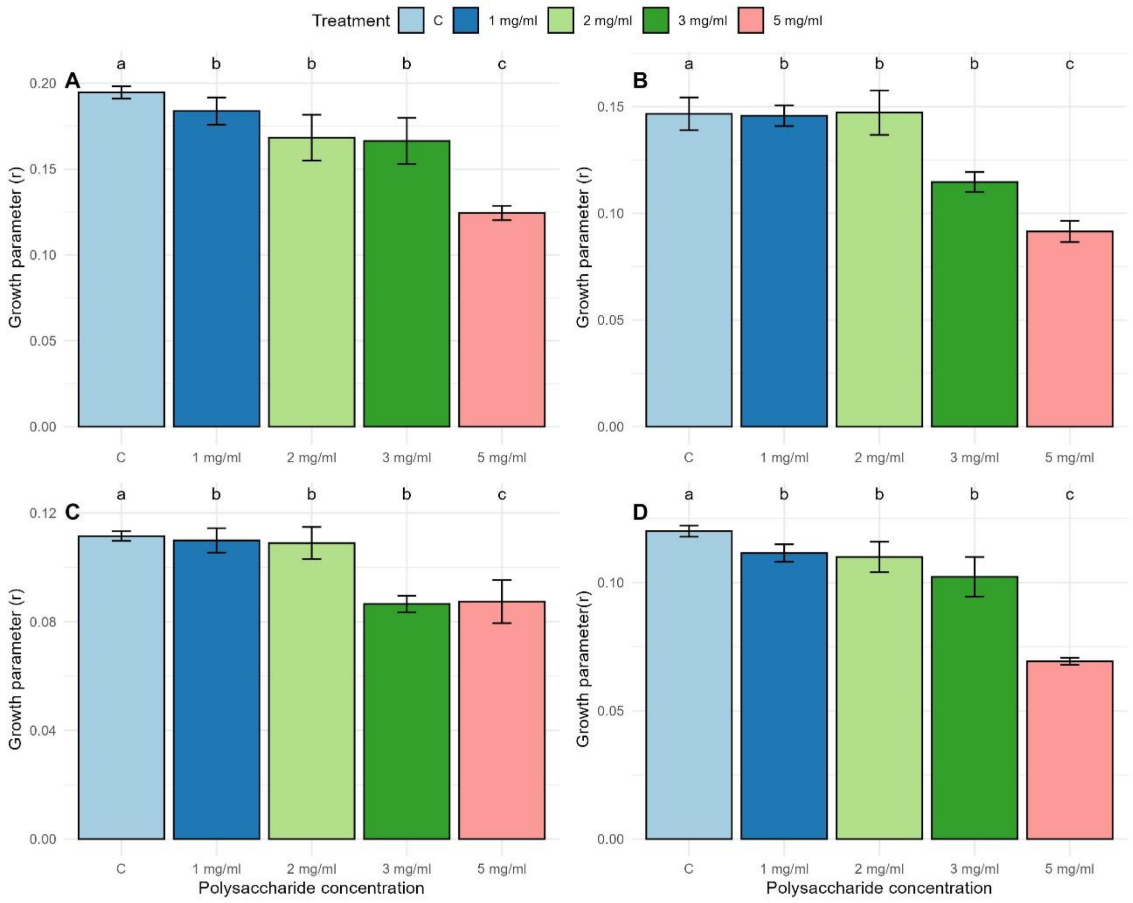

Figure S12. Effect of seaweed polysaccharides on the growth parameter (r) of A) *C. albicans*, B) *C. auris*, C) *Cr. bacillisporus*, and D) *Cr.deuterogatti* at different concentrations (1, 2, 3 and 5 mg mL^{-1}). C: control. Bars represent the mean ± standard deviation. Different letters indicate significant differences among treatments according to Tukey's post hoc test ($p < 0.05$).

Table S6. One-way ANOVA results for the growth inhibition of the different yeast species exposed to different concentrations of seaweed polysaccharides. Df: degrees of freedom. Sum Sq: sum of squares. Mean Sq: mean squares. F value: F statistic value.

Species	Df	Sum Sq	Mean Sq	F value	p.value
C. albicans	3	4794	1598	72.69	**<0.001**
	8	176	22		
C. auris	3	10357	3452	106.8	**<0.001**
	8	259	32		
C. bacillisporus	3	2188.9	729.6	6.381	**0.016**
	8	914.7	114.3		
Cr. deuterogatti	3	4486	1495.3	23.52	**<0.001**
	8	509	63.6		

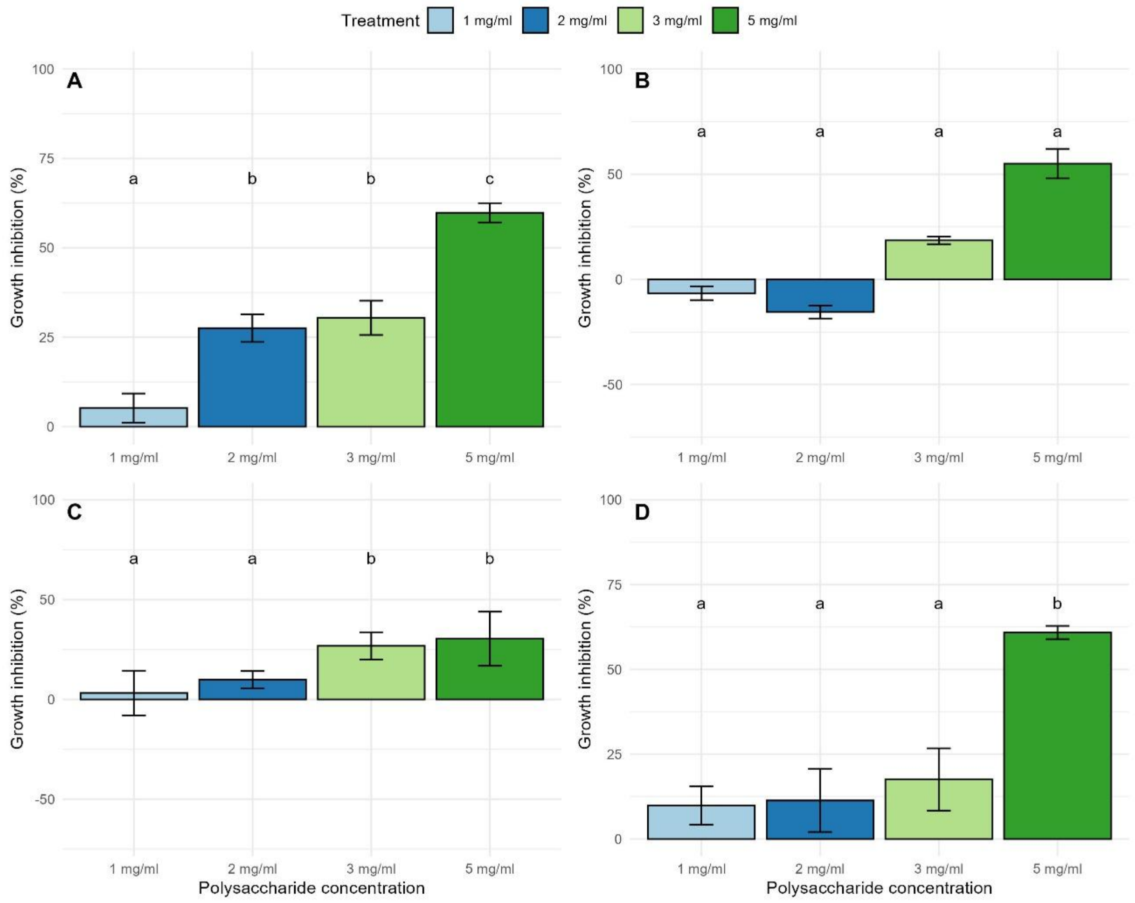

Figure S13. Effect of seaweed polysaccharides on growth inhibition (%) of A) *C. albicans*, B) *C. auris*, C) *Cr. bacillisporus* and D) *Cr. deuterogatti* at different concentrations (1, 2, 3 and 5 mg mL). Bars represent the mean ± standard deviation. Different letters indicate significant differences among treatments according to Tukey's post hoc test ($p < 0.05$).

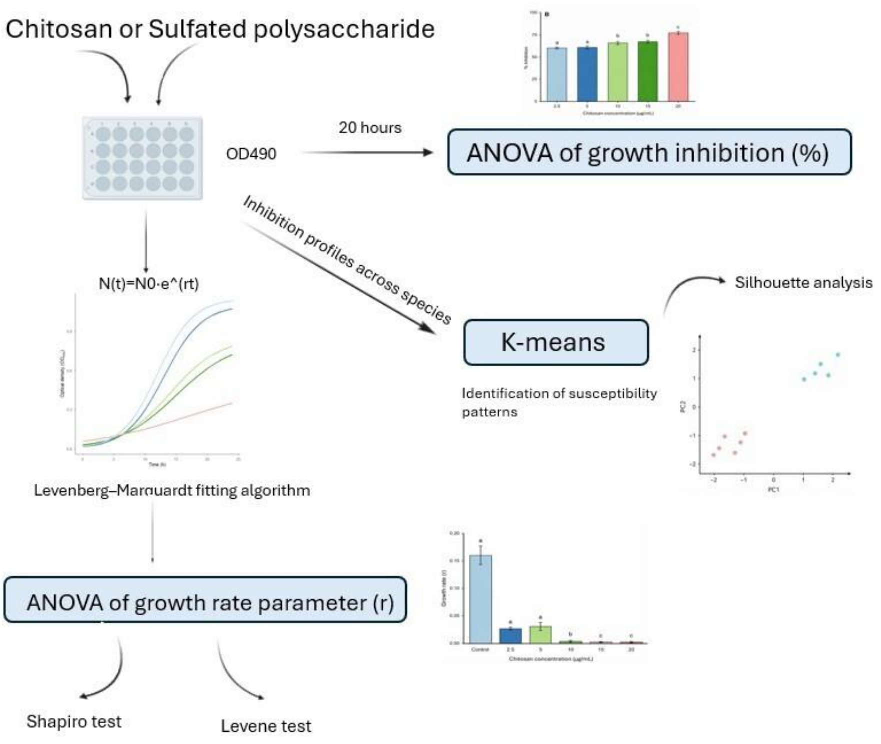

Figure S14. Schematic representation of the statistical workflow applied to evaluate the antifungal activity of chitosan and sulfated polysaccharides against yeast species. Growth kinetics were monitored over 24 h using OD490 measurements and fitted to an exponential growth model using the Levenberg–Marquardt algorithm to obtain the growth rate parameter (r), which was subsequently analyzed by ANOVA. In parallel, growth inhibition percentages at 20 h were calculated and analyzed by ANOVA. Species susceptibility patterns were further explored through K-means clustering based on inhibition profiles across concentrations, with the optimal number of clusters determined using silhouette analysis.
